# Pulsatilla in Inflammations of the Testis

**Published:** 1882-03

**Authors:** G. Frank Lydston

**Affiliations:** 137 East Madison St., Chicago


### ARTICLE IV.

### PULSATILLA IN INFLAMMATIONS OF THE TESTIS. By G. FRANK LYDSTON, M.D.

Although the use of pulsatilla in the treatment of disease has been chiefly confined to the homœopaths, attention has been called to its efficacy, particularly in inflammatory affections of the testis, by several members of the regular profession, Dr. Piffard having especially commended it.

A short time since, having a hospital service in which there were abundant opportunities to test its efficacy, I began its use in such cases. I had previously employed the ordinary methods of treatment, such as applications of ice, hot fomentations, tobacco poultices, etc., thus having ample scope for the comparison of the results derived from the different procedures. The results of the remedy were very gratifying, and in most instances, whether given alone or in combination, superior to the ordinary measures for relief of the above inflammations. The records of the cases so treated at that time I did not retain, but they contained a large number of cases. I have been fortunate enough to have a series of five pronounced cases within the last few months, the histories of which I have, and will recount, hoping that they may prove of interest.

Case I.—*Gonorrhœal epididymitis.*—Patient is a plethoric subject, æt. 35; stated that he contracted a severe gonorrhœa some three weeks previously, which ran the usual course, until two days before consultation, when the right testicle began to swell and became very painful, the urethral discharge having ceased. He complained greatly of dragging pain along the cord The epididymis of the affected side was extremely swollen and of almost stony hardness, and the tunica vaginalis greatly distended . the tenderness on pressure was very marked. He was ordered a mercurial cathartic, and tr. pulsatillæ in 10 m. doses, every two hours. The testes were supported upon oakum. On the following morning the pain had entirely ceased and the tenderness was perceptibly less. There was no change, however, in the bulk of the testicle, although the fluid in the tunica vaginalis seemed less transparent. At the end of twenty-four hours the pulsatilla was stopped. There was no recurrence of the pain, and on the fourth day the tenderness was so far reduced that strapping was admissible, and was continued until the organ had regained its usual size.

Case II.—*Rheumatic orchitis.*—Patient, a carpenter; æt. 24. Denied having had venereal disease of any description, and stated that he contracted his present trouble by sitting upon a cold stone step for some time, he having on thin pantaloons. The pain was especially severe, and was accompanied by considerable nausea and fever. There was also some tenderness and swelling of the ankle joints. The body of the testis was swollen and tender; the epididymis somewhat enlarged, with slight effusion into the tunica vaginalis. He was given a saline cathartic and put upon tr. pulsatillæ m. v. every hour. In twenty-four hours the pain which had been so severe, that I had thought seriously of subcutaneous incision of the testis, had considerably abated, and on the third day had disappeared, leaving, however, some tenderness and no appreciable change in the swelling. Hot fomentations and poultices were now ordered and continuously applied, until the induration had disappeared.

Case III.—Epididymitis from passage of sounds for relief of stricture and gleet, of long standing. Patient, engineer; had had gleet for over a year, and had previously had a number of attacks of gonorrhœa. On the day following a somewhat prolonged instrumentation of the urethra, pain and great tenderness of the left testicle developed. The same treatment was adopted as in the preceding case, with, however, in addition, x minims of fl. ex. jaborandi every hour, he having considerable fever and complaining greatly of thirst. On the third day pain had entirely ceased and poultices were ordered.

Case IV.—*Gonorrhœal epididymitis.*—History of previous attack in same testicle, which laid the patient up for four weeks. The same line of treatment was adopted as in previous cases, and with marked success—the pain ceasing in twenty-four hours, and the gentleman being at his work again on the eighth day, the testicle, however, being strapped to produce resolution of the swelling and support the organ, thus preventing a relapse and enabling the patient to attend to his business, which compelled him to be upon his feet a great deal of the time.

Case V.—Somewhat similar to preceding case. Swelling and intense pain of neuralgic character in both the inflamed and sound organs, this pain having severe exacerbations shooting along spermatic cord and down the inner aspect of thighs. There was also considerable pain in the back. Fifteen minims of the tr. pulsatillæ were given every second hour, with the usual preliminary cathartic. In forty-eight hours the pain was markedly diminished, there being, however, some increase of the swelling. The remedy was stopped on the third day and poultices applied. There was a slight recurrence of the pain on the fifth day, which was again readily controlled, in a few hours, by the pulsatilla.

From observation of the action of pulsatilla upon the above cases and others, which, though carefully noted as to the effects of the remedy, were not recorded, I have been led to the conclusion that it is of great value. The drug has long borne the opprobrium of being a homœopathic preparation, and has been highly recommended by the small-pill fraternity; they, however, have administered it only in infinitesimal doses, in which it is obviously impossible to obtain its physiological effects, so that it would be practicable to determine whether their so-called beneficial results were due to their small doses of the drug or were really the natural course of the affection,. they never having applied rational measures of treatment, and being consequently devoid of data for comparison. They do not give any explanation of its action, but speak in a vague manner of its specific effect upon the mucous membranes, the female generative organs and the cerebro-spinal axis. It is probable that its effects are manifested principally through its influence upon the nervous system. It seems to have a specific action upon the testis itself, as evidenced by the speedy relief of pain and tenderness, although it has no effect upon the induration of the inflamed organ, and does not appreciably diminish the serous effusion attendant upon such cases. The beneficial effects of the drug in gonorrhœal epididymitis may possibly be due in some measure to its action upon the inflamed urethral tract, which constitutes the source of most of the cases of that disease.

I can not reconcile my observations to those of Dr. Piffard, who in some of his earlier investigations of the subject found that five minim doses of tr. pulsatillæ aggravated epididymitis, while the same drug, in doses of one-tenth of a minim every three hours, rapidly cured the disease. The doctor evidently has great faith in infinitesimals.

I am not aware to what extent regular practitioners have investigated the action of pulsatilla, nor to what extent it has been written upon, but I trust that it may receive a fair trial, and if it be found to be as valuable as I have been led to believe it, that it may enter more extensively into the pharmacopœias of legitimate physicians, thus rescuing a useful drug from the obscurity of homœopathic practice.

137 EAST MADISON ST., CHICAGO.

